# Ebulin from Dwarf Elder (*Sambucus ebulus* L.): A Mini-Review

**DOI:** 10.3390/toxins7030648

**Published:** 2015-02-25

**Authors:** Pilar Jiménez, Jesús Tejero, Damián Cordoba-Diaz, Emiliano J. Quinto, Manuel Garrosa, Manuel J. Gayoso, Tomás Girbés

**Affiliations:** 1 Nutrition and Food Science, Faculty of Medicine, and CINAD (Center for Research in Nutrition, Food and Dietetics), University of Valladolid, Valladolid E-47005, Spain; E-Mails: pilarj@bio.uva.es (P.J.); jesus.tejero@uva.es (J.T.); equinto@ped.uva.es (E.J.Q.); 2 Pharmacy and Pharmaceutical Technology, Faculty of Pharmacy, and IUFI (Institute of Industrial Pharmacy), Complutense University of Madrid, Madrid E-28040, Spain; E-Mail: damianco@farm.ucm.es; 3 Cell Biology, Histology and Pharmacology, Faculty of Medicine, and INCYL (Institute of Neurosciences of Castile and Leon), University of Valladolid, Valladolid E-47005, Spain; E-Mails: garrosa@med.uva.es (M.G.); gayoso@med.uva.es (M.J.G.)

**Keywords:** *Sambucus ebulus* L., blossoms, lectin, ebulin, ricin, ribosome-inactivating protein

## Abstract

*Sambucus ebulus* L. (dwarf elder) is a medicinal plant, the usefulness of which also as food is restricted due to its toxicity. In the last few years, both the chemistry and pharmacology of *Sambucus ebulus* L. have been investigated. Among the structural and functional proteins present in the plant, sugar-binding proteins (lectins) with or without anti-ribosomal activity and single chain ribosome-inactivating proteins (RIPs) have been isolated. RIPs are enzymes (E.C. 3.2.2.22) that display *N*-glycosidase activity on the 28S rRNA subunit, leading to the inhibition of protein synthesis by arresting the step of polypeptide chain elongation. The biological role of all these proteins is as yet unknown. The evidence suggests that they could be involved in the defense of the plant against predators and viruses or/and a nitrogen store, with an impact on the nutritional characteristics and food safety. In this mini-review we describe all the isoforms of ebulin that have to date been isolated from dwarf elder, as well as their functional characteristics and potential uses, whilst highlighting concern regarding ebulin toxicity.

## 1. Introduction

From an archaeological point of view, the most widely studied elders were the European species *Sambucus ebulus* L. and *Sambucus nigra* L. Remains of pollen, seeds/fruits and charcoals of *S. ebulus* L., known also as danewort and European dwarf elder, have been found at a Bronze Age archaeological site in Tuscany (Italy) [1], at a Neolithic site in the French Alps (Bozel-Savoie, France) [2] and at the Durankulak-3 site the Black Sea coast and north-eastern Bulgaria [3]. Dwarf elder grows in the wild and abundantly in many temperate areas and requires no care. Therefore, its use in human subsistence may be surmised. However, since the plant is toxic, it probably underwent a certain culinary treatment in an attempt to reduce its toxicity. 

A broad range of medicinal applications of dwarf elder has been reported [4]. Among them, the improvement of lipid profile, antimicrobial, antiulcerogenic, antiparasitic, anti-inflammatory applications, and wound healing activities have been cited [5,6,7,8,9,10]. These effects must be due to the presence of chemical compounds with pharmacological activity. For instance, polyphenolic substances have been associated with antioxidant action [11,12]. The cyan color of the ripe dwarf elder fruit is due to several anthocyanidins such as cyanidin-3-*O*-sambubioside, cyanidin-3-sambubioside-5-glucoside, cyanidin-3-*O*-glucoside, and cyanidin-3,5-diglucoside [13]. Cyanidin-3-*O*-glucoside is especially important since it has been found to inhibit the growth of cancer cells and tumor xenographs in immune-deficient mice [14].

Here we review the literature on ebulin, a ribosome-inactivating protein found in *S. ebulus* L., considering the isoforms found in the different tissues, the structural studies carried out on the ebulin present in leaves (ebulin l), and the consequences of its enzymatic activity such as its use in targeted therapy as a toxic moiety of immunotoxins and conjugates, and the *in vivo* toxicity caused by its ingestion in large amounts.

## 2. RIPs Isolated from *S. ebulus* L.

In addition to the general structural and enzymatic protein characteristics of plant species, *Sambucus* contains bioactive proteins like single- and two-chain lectins and ribosome-inactivating proteins (RIPs) [15,16,17]. Lectins are proteins that bind sugars, and RIPs are enzymes (E.C. 3.2.2.22) that carry out hydrolysis of the N-glycosidic bond linking adenine 4324 (A_4324_) to the ribose-phosphate backbone of the 28S rRNA in the rabbit reticulocyte ribosome [18,19]. This adenine is located in a highly conserved loop known as the α-sarcin/ricin loop (SRL), which is responsible for the interaction of the eukaryotic elongation factor 2 (eEF-2) with the mammalian ribosome [19], and the elongation factor G (EF-G) in *Escherichia coli* [20,21]. RIPs have also been described as antiviral proteins [22]. In fact, one of the first RIPs shown to display antiviral activity was the *Phytolacca americana* antiviral protein (PAP) [23]. RIPs have been classified in types 1, 2 and 3 depending on their structure [15,16,17]. Type 1 RIPs are single chain (A chain), and contain the enzymatic activity. Type 2 RIPs are two or four chain (A-B or [A-B]_2_) proteins in which the A chain is the enzyme and the B chain is a lectin. Type 3 RIPs are a special single chain type of RIPs found only in *Poaceae* [16,17]. Among the RIPs of *S. ebulus* L. type 1 RIPs, like ebulitins α, β and γ, and type 2 RIPs, such as different forms of ebulin, have been found.

## 3. Isoforms of Ebulin

Dwarf elder has a number of different isoforms present in variable amounts in different parts of the plant:

### 3.1. Ebulin l

Black and dwarf elders are the ones so far most widely studied in this context. The first RIP isolated from dwarf elder was ebulin l [24]. Ebulin l is present in the leaves of dwarf elder as a two-chain (A-B) protein of apparent Mr of 56,000, with an A chain (apparent Mr of 26,000) which contains the *N*-glycosidase activity, and a B chain which displays lectin activity specific for d-galactose, both chains being linked by a disulphide bond. Ebulin l was isolated by affinity chromatography through acid-treated Sepharose 6B and separated from a two chain lectin of the B-B type, with an apparent Mr of 68,000 that we named SELld, by molecular exclusion through Superdex 75. Ebulin l exhibits great inhibitory activity on mammalian protein synthesis, carried out in the presence of a reductant, with IC_50_ (a concentration that gives 50% inhibition) values of 8.5, 15 and 5 ng/mL for rabbit reticulocytes, rat liver and rat brain cell-free systems, respectively. By contrast, ebulin l does not inhibit protein synthesis in plant and bacterial cell-free systems. As will be seen below, ebulin l is about 10,000 times less toxic for mammalian cultured cells than ricin. This *in vitro* activity is reflected *in vivo*, and accordingly ebulin l has an intraperitoneal (i.p.) LD_50_ for mice close to 2.5 mg/kg of body weight, in contrast with ricin, whose toxicity depends on the administration route, with values of 0.023 and 0.0075 µg/kg of body weight for the i.p. and intravenous (i.v.) administrations, respectively [25]. This difference in toxicity led us to consider ebulin l together with nigrin b, an equivalent related RIP isolated from *S. nigra* L. (black elder) [26] as non-toxic type 2 RIPs [15].

### 3.2. Ebulin r1 and r2

Two isoforms of ebulin, ebulin r1 and r2, were isolated from rhizomes of dwarf elder [27]. They were isolated by affinity chromatography through acid-treated Sepharose 6B, followed by Superdex 75HiLoad chromatography, and finally Mono-Q chromatography. Ebulin r1 eluted before ebulin r2 from Mono-Q column chromatography with a linear gradient of 0–0.3 M NaCl. This affinity chromatography protocol allowed isolation of the two isoforms of ebulin together with the previously described tetrameric lectin known as SEA [28], and two new lectins that we named as SEA II-1 and SEA II-2. The apparent molecular masses of ebulins r1 and r2 were 26,000 for the A chains and 30,000 for the B chains. Ebulin r1 and r2 display a strong inhibitory activity on protein synthesis carried out by cell-free systems from mammalian tissues (IC_50_ of 1.7 ng/mL for rabbit reticulocyte lysates). This effect is dependent on the *N*-glycosidase activity on the ribosomes. Ebulins r1 and r2 are different proteins, as revealed by ELISA analysis [27] and by the translational inhibitory activity [29]. Therefore while ebulin l is inactive in the native unreduced form (protein synthesis carried out in the absence of a reductant), both rhizome ebulins are active. Ebulin r2 is as active as the reduced ebulin l and 100 times more active than the unreduced ebulin r1. In addition, it was found that ebulin l, r1 and r2 trigger a strong multi-depurination activity on RNA and DNA that is comparable to ricin [29].

### 3.3. SEA I

This protein was described as a lectin which interacts with sialic acid like SNAI and, therefore, it was isolated by affinity chromatography through fetuin-agarose followed by ion-exchange on Mono-Q, and gel filtration through Superose 12HR [30]. Its affinity for galactose allowed its isolation by the same procedure as the one used for ebulin l [24]. Polyacrylamide gel electrophoresis with sodium dodecyl sulfate revealed a homogeneous protein with an apparent Mr of 140,000. With 2-mercaptoethanol as a reductant, 5 protein bands with a molecular weight of between 30,000 and 35,000 were revealed. MALDI-TOF mass spectrometry analysis of native protein revealed an average molecular weight at *m/z* 135,630. Dithiothreitol-reduced protein gives two main peaks, ranging between 33,890 and 35,893 for the A chain and the B chain, respectively. These values are consistent with the tetrameric structure (A–B)_2_ as suggested previously [28]. In addition, SEA contains *N*-linked polysaccharide chains. SEA has been proven to be a true RIP which not only depurinates the ribosomal RNA but also has an effect on DNA from cultured COLO 320 cells. Histological analysis showed that SEA binds to glycoproteins and glycolipids on the plasma membrane and accumulates in intracellular vesicles. Fluorescence-labeled SEA binds to the goblet cells present in the villi of the small intestine epithelium. These cells are specialized in the synthesis and accumulation of mucin, whose oligosaccharide chains often terminate in sialic acid [31].

### 3.4. Ebulin f

Fruits of dwarf elder contain an isoform of ebulin l that we called ebulin f [32], and which was isolated by the same procedure as ebulin l [24]. The use of affinity chromatography through acid-treated Sepharose 6B allowed the simultaneous isolation of SEA and a homodimeric d-galactose lectin named SELfd which were separated through Superdex 75HiLoad. The apparent molecular mass values for ebulin f were 30,000 and 34,000 for the A and B chains, respectively, and 64,000 for the native protein. MALDI-TOF mass spectrometry analysis of the native protein revealed a molecular mass at *m/z* of 58,904 [33]. It is noteworthy that the procedure also allowed the isolation of protein material with apparent Mr higher than 100,000, which was shown to be formed by polymerized ebulin mixed with polymerized lectin. Polymerization was seen to take place through disulphide bonds, which are reduced by 2-mercaptoethanol and newly formed by elimination of the reductant by dialysis. Ebulin f displays a very high affinity for mucin and a sialo-mucin. Ebulin f and the lectin SELfd show a differential sensitivity to digestion by a simulated gastric fluid [33]. Native SELfd was unmodified by pepsin in an incubation protocol of 60 min at 37 °C. By contrast, ebulin f started to degrade after just 15 min of incubation, and was almost completely hydrolyzed after 60 min. Mass spectrometry analysis revealed that certain tryptic peptides derived from both ebulin f and SELfd share amino acid sequence identity with tryptic peptides obtained from the allergen Sam n1 isolated from elderberry [34]. This raised the hypothesis, which is being currently investigated, that the proteins related structurally with Sam n1 and ebulin would probably make up a new family of allergens.

Ripe dwarf elder fruits may be useful as a source of nutraceuticals. They are a rich source of phenols and antioxidant compounds [11,13]. However, they coexist with two-chain lectins, named SELfd and ebulin f, which are resistant to pepsin digestion [35]. Since, as will be indicated below, ebulin f displays a significant oral toxicity in mice, it is important to reduce the influence of ebulin f without affecting its antioxidant capacity. A short heating of ripe fruit extracts only reduced the content of total phenols and the antioxidant and free-scavenging activities by up to 13.5% but completely sensitized ebulin f to pepsin [36].

### 3.5. Ebulin blo

Dwarf elder blossoms also contain an isoform of ebulin, which we named ebulin blo, together with a homodimeric lectin we called SELblo [36]. MALDI-TOF mass spectrometry analysis of the native protein revealed a molecular mass at *m/z* of 63,225. Tryptic fingerprinting indicated that, as in the case of ebulin l, ebulin blo contains sequences present also in the Sam n1 allergen. As will be shown below, ebulin blo is toxic when administered to mice by gavage.

## 4. Molecular Cloning and Structure of Ebulin l

Molecular cloning and the amino acid sequence of ebulin l were carried out from RNA using young leaves collected in early summer [37]. The cDNA for ebulin l contains an open reading frame of 1692-bp that encodes a polypeptide of 564 amino acids. After cleavage of the signal peptide the resulting polypeptide chain contains the *N*-terminal amino acid sequence of both the A and B chains. An analysis of the amino acid sequence revealed that identities of both ebulin l chains with those of ricin are 34% for A chains and 48% for B chains.

Highly purified ebulin l was crystalized in two crystal systems, namely, orthorhombic and trigonal [37]. The crystals were analyzed at 2.8 Å by X-ray irradiation technique. Data collected indicate that ebulin l A chain has the same positioning of the key amino acids required for the inhibition of translation as the ricin A chain. An analysis of sugar binding indicates that the ebulin crystals bind lactose and galactose to the subdomain 1α similarly to ricin. By contrast, the subdomain 2γ of ebulin B chain binds only galactose. This may be the reason why ebulin has a lower affinity for a dense matrix of galactose than ricin [37]. The consequence of this different means of binding sugars in the subdomain 2γ is that ebulin has less ability to bind galactosides as compared with ricin and, therefore, this could be the reason for the reduced toxicity for cultured animal cells and *in vivo* toxicity for mice.

## 5. *In planta* Developmental Tissue Occurrence of Ebulin

A description has been given of ebulin occurrence in fruits; leaves and blossoms depending of the stage of development. In fruits; ripening led to a significant reduction in the concentration of type 2 RIPs and the structurally-related lectin SELfd; green dwarf elder fruits contain approximately 16 mg/kg of wet weight of ebulin f; while maturation reduces it to traces [32]. This is quite similar to the evolution of ebulin l in leaves of dwarf elder [38]; and nigrin f in fruits of elderberry [39]. Polymerization of ebulin f takes place only in green fruits when its concentration is the maximum possible; and it disappears upon ripening. The largest concentration of ebulin l is found in shoots of 1–2 mm in length; while it is minimal in senescent leaves. Furthermore; such an accumulation is inverse to the accumulation of the structure-related lectin SELld [24]. At present we do not know the biological role of these type 2 RIPs; we hypothesize that ebulin; at least in leaves; could be an anti-nutrient for predator insects; as previously reported for other type 2 RIPs [40]; at a moment in which the leaves are growing and require all the energy for such a process.

## 6. Use of Ebulin in Targeted Therapy

As mentioned above, both type 2 RIPs of *Sambucus*, ebulin and nigrin, are much less toxic to cultured cells and *in vivo* than ricin. Nonetheless, at the ribosomal level, they are as toxic as ricin. The reason for this difference seems to be the internalization route of these proteins. Nigrin b, a type 2 RIP similar to ebulin l [26], follows a pathway that goes from endosomes to lysosomes, where they are degraded and the inactive products expelled from the cell [41]. The accumulation of these RIPs in endosomes would favor concentration dependent non-specific translocation to the cytosol, thus allowing transport to the ribosomes.

The intracellular pathway of ricin in animal cells follows the trans-Golgi rough endoplasmic reticulum and further translocation to the cytosol [42,43]. Even tough we do not have a detailed molecular description on ebulin intracellular traffic, we hypothesized that the differences between native and engineered type 2 RIPs are in the ability of the B chain to bind to cells, uptake and membrane translocation [44]. It has been shown that ebulin l has a low affinity for galactose as compared with ricin, due to a change in the structural disposition of the 2γ-subdomain of the ebulin B chain respect to ricin which reduces the ebulin ability to bind galactosides [37]. We believe that such differential affinity for galactosides would determine its intracellular fate and possibly its cytotoxicity. Our data, together with the data on the traffic of a similar type 2 RIP like, nigrin b [41] suggests that ebulin probably does not reach the endoplasmic reticulum and therefore does not suffers retro-translocation to the cytosol. Nonetheless, an alternative explanation of the different cytotoxicity of ricin and ebulin could be related with the unfolded protein response (UPR) responsible of the proteasome-dependent protein degradation that is inhibited by ricin to attenuate another target able to enhance its cytotoxicity [45,46]. Our hypothesis is that most probably ebulin is unable to inhibit UPR and the consequence would be the degradation of ebulin before to reach the ribosomes.

The low toxicity of ebulin *in vivo* and in cultured mammalian cells compared to ricin prompted us to use it for the construction of immunotoxins and conjugates for the targeted therapy [47]. An immunotoxin has been constructed containing ebulin l and anti-human CD105 monoclonal antibody 44G4 [48]. The procedure used was conjugation of both proteins with *N*-succinimidyl-3-(2-pyridildithio)-propionate (SPDP), which links them together with a disulphide bond. The coupling reaction produces a mixture of conjugates that, upon purification to species with two molecules of ebulin per molecule of antibody, retains a substantial protein synthesis inhibitory activity on human CD105 expressing cells, with an IC_50_ ranging from 3 × 10^−10^ to 4 × 10^−9^ M. This is in contrast with the effects of unconjugated ebulin l, which in the same conditions has 3 logs lower toxicity [47].

Conjugates of ebulin with non-antibody proteins have also been prepared. A conjugate of ebulin l with human transferrin has also been constructed, retaining both targeting ability to transferrin-receptor expressing cells and inhibition of cell protein synthesis depending on targeting. Among several linkers assayed, the best results were obtained with SPDP [49].

## 7. *In Vivo* Toxicity of Ebulin f

Despite the low toxicity of ebulin isoforms *in vivo* and in cultured animal cells as compared with ricin, they exhibited toxicity when large amounts (5 mg/kg body weight) were injected either intravenously (i.v.) or intraperitoneally (i.p.). Thus, mice treated with lethal doses of ebulin f show histopathological changes in the stomach, intestines, lungs and heart [50].

The gastric mucosa is scarcely damaged, showing no more than a mild loss of mucocytes. The mucosa of the small intestine, however, appears considerably affected as a consequence of ebulin administration. The villi are shorter and less numerous, while Lieberkühn’s crypts appear quite atrophic. Damaged small intestine crypts are filled with dead cells and remains of cells from the crypt walls [51]. The villi appear edematous and undergo derangement, enterocytes being subsequently replaced by flattened epithelial cells. An increase in the number of apoptoses can be observed in the intestinal epithelium, particularly in the transit amplifying compartment (TAC), leading to a diminished turnover rate and consequently to the disappearance of many crypts and villi. The *lamina propria* is also damaged, losing its normal association with the epithelium. The failure of this epithelial barrier could very well be the cause of the severe hemorrhages seen in the small intestine when the autopsies of these treated animals are performed.

The large intestine is also altered but to a lesser extent. Lieberkühn’s crypts preserve their general cytoarchitecture, cell loss occurring mainly in enterocytes since goblet cells seem to be more resistant. Damaged cells appear mainly in the middle third of the crypt, the number of apoptoses observed in the large intestine being fewer in comparison with the small intestine [51]. The lungs appear congestive and in some animals there is alveolar hemorrhage [50]. The cardiac muscle appears a poorly cohesive tissue, including cardiomyocytes with signs of weakness and dehiscence among them [50].

## 8. Concluding Remarks

Ebulin is a type 2 RIP of dwarf elder that occurs in leaves, fruits, rhizomes and blossoms in isoforms and differs in molecular mass and activity on nucleic acids. Its molecular activity on the ribosome is the same as that of ricin but, similarly to nigrin b of elderberry, is several times less toxic than ricin when injected parentally. Ebulin and the related lectins share some domains of amino acid sequences with the allergen Sam n1 isolated form elderberry. This has led us to hypothesize that these proteins would represent a new family of allergens present in *Sambucus*. Further work is needed to examine intracellular trafficking and the mechanisms whereby ebulin triggers the toxic effect, as well as to investigate the toxicity due to ebulin of raw preparations of dwarf elder fruits added maliciously to foods, and whether its neutralization by food matrices and liquids could be feasible. The effects of ebulin require more research effort, especially in the development of analytical procedures for its detection and treatment.
